# Behavioral Economics Incentives to Support HIV Treatment Adherence (BEST): Protocol for a randomized controlled trial in Uganda

**DOI:** 10.1186/s13063-019-3795-4

**Published:** 2020-01-03

**Authors:** Sebastian Linnemayr, Chad Stecher, Uzaib Saya, Sarah MacCarthy, Zachary Wagner, Larissa Jennings, Barbara Mukasa

**Affiliations:** 1 RAND Corporation, Santa Monica, USA; 2 Arizona State University, Phoenix, AZ USA; 3 Johns Hopkins University, Baltimore, MD USA; 4 Mildmay Uganda, Kampala, Uganda

**Keywords:** HIV/AIDS, Behavioral Economics, Incentives, Lottery, Uganda, Global Health, Randomized Controlled Trial, Present Bias

## Abstract

**Background:**

Many HIV-positive patients do not appropriately adhere to their antiretroviral medication (ART). This leads to higher viral loads and greater probability of HIV transmission. Present bias—a tendency to give in to short-term temptations at the expense of long-term outcomes—is a potential driver of low adherence. In this study we test a novel intervention rooted in behavioral economics that is designed to overcome present bias and increase ART adherence.

**Methods/design:**

We will enroll 330 HIV-positive patients at Mildmay Hospital in Kampala, Uganda, into a 2-year randomized controlled trial. Participants will be randomized to one of three groups. The first intervention group (T1, *n* = 110) will be eligible for small lottery prizes based on timely clinic visits and demonstration of viral suppression. Group 2 (T2, *n* = 110) will be eligible for the same lottery prizes conditional on high adherence measured by a medication event management system (MEMS) cap. The control group (*n* = 110) will receive the usual standard of care. Adherence will be measured continuously throughout the intervention period and for 12 months post-intervention to evaluate effect persistence. Surveys will be conducted at baseline and then every 6 months. Viral loads will be measured annually. Primary outcomes are whether the viral load is detectable and MEMS-measured adherence. Secondary outcomes are the log-transformed viral load as a continuous measure and a binary measure for whether the person took at least 90% of their ART pills.

**Discussion:**

Our study is one of the first to investigate the effectiveness of lottery incentives for improving ART adherence, and in addition, it compares the relative efficacy of using electronically measured adherence versus viral load to determine lottery eligibility. MEMS caps are relatively costly, whereas viral load testing is now part of routine clinical care in Uganda. BEST will test whether directly incentivizing viral suppression (which can be implemented using readily available clinic data) is as effective as incentivizing electronically measured adherence. Cost-effectiveness analyses of the two implementation modes will also be performed.

**Trial registration:**

ClinicalTrials.gov, NCT03494777. Registered on 11 April 2018.

## Background

Treatment adherence is critical to the success of antiretroviral treatment (ART) and is largely determined by behavior. Over 1.3 million Ugandans are infected with HIV and prevalence is still over 6% [1]. ART has improved the life expectancy of people living with HIV/AIDS in Uganda dramatically, and a scaling up of treatment has resulted in over 72% of these Ugandans receiving ART [2–8]. Yet the success of these drugs is dependent on high *lifelong* medication adherence to achieve optimal clinical outcomes, such as slowing the progression to AIDS, lengthening survival, sustained viral suppression, and prevention of drug resistance and loss of treatment options [9–14]. Moreover, ART adherence in Uganda may be lower than previously assumed [15] and be declining over time [16, 17]. Both structural (e.g., drug availability) and practical (e.g., distance to clinic and treatment fees) adherence barriers have been investigated [18–20], yet patient behavior has emerged as a key factor for adherence [21].

Newer, simpler regimens are also plagued by low adherence, and are often not available in resource-poor countries. These ART regimens are more forgiving [22–24], but result in only marginally better adherence [25]. Adherence remains key to the success of ART [26–28], and at least 80–85% adherence is needed to sustain viral suppression and prevent drug resistance [22–24]. Despite the importance of adherence and simpler regimens, mean ART dose-taking adherence (percentage of prescribed doses taken) typically ranges from 60 to 80% when measured electronically, and only 30–60% of patients achieve 85% adherence [29–31]. In addition, these simpler regimens are often not available in sub-Saharan Africa and other resource-constrained environments.

A growing body of literature suggests that *motivation* is a strong predictor of adherence, yet maintaining high motivation is likely to be challenging for those who have been on ART for many years [32]. Clients who have been on ART for many years have unique challenges in sustaining good adherence, in particular treatment fatigue, or the “decreased desire and motivation to maintain vigilance in adhering to a treatment regimen among patients prescribed long-term protocols” [32]. While treatment fatigue is increasingly becoming recognized as an important problem, currently no behavioral interventions have been developed to treat it [32]. Recent evidence from Uganda indicates that clients take drug holidays when they feel overwhelmed by the daily task of taking their drugs lifelong [33], putting the motivation needed to fight treatment fatigue in the foreground. Therefore, targeting motivation through rewards for healthy behaviors may be particularly appropriate for treatment-mature clients.

Behavioral economics can explain why people do not always adhere to healthy behaviors and why incentives may be necessary to achieve desired health outcomes. People often fail to act in their own self-interest and commonly behave in ways they afterwards regret, such as overeating or smoking [34, 35]. Behavioral economists refer to this phenomenon as *present bias*, e.g., a tendency to give in to short-term temptations at the expense of long-term outcomes [36]. This phenomenon is particularly likely for health behaviors for which the benefits occur in the distant future and the absence of disease is largely invisible and becomes apparent only once health has deteriorated significantly, as is the case for HIV [37]. Prior work shows that present bias is indeed common among HIV clients in Uganda and that clients displaying present bias were 13 percentage points less likely to show adherence above 90%. Present bias, therefore, seems to be a significant predictor of subsequent adherence [38].

For clients on ART, the benefits of adhering occur far into the future, and as a result, present-biased clients may fail to adhere. We hypothesize that rewards for high adherence in the present address present bias, which will overcome treatment fatigue and increase adherence. Evidence from our previous study indicates that clients receiving lottery prizes were more likely to show adherence above 90% than those in the control group. Offering participation in a prize draw also offers a sense of fun and entertainment to clients who are mostly very poor and face daily struggles. Our clinical colleagues often talk about how small things bring enjoyment, laughter, or a smile to the face of clients. A participant in an exit focus group discussion of a previous study stated that: “There is little joy in the life of us [HIV clients], and coming to the clinic and having the chance to win a prize really brings me a lot of happiness, even if I don’t always win” (Male adult client).

Rewards have improved a number of health behaviors effectively (including HIV-related ones), and emerging research in behavioral economics suggests ways of increasing the effectiveness of incentives. A review study by Kane et al. [39] found that economic incentives increased attendance at HIV and sexually transmitted disease educational sessions, condom purchases, and participation in HIV counseling and testing. Behavioral economics suggests that frequent small nudges can address present bias and alter behavior effectively. Rather than focus on the magnitude of the prize as in the traditional literature, behavioral economics suggests that the way prizes are given out—and at what time intervals—significantly determines their effectiveness. Prize draws leverage the bias of overestimation of small probabilities (leading individuals to participate in the draw because they overestimate their chance of winning) and thereby also increase salience (frequent prizes keep a behavior high on a person’s mental priority list) [40, 41]. A number of studies have documented the positive impact of lotteries in shifting complex health behaviors, such as breastfeeding, losing weight, as well as preventing obesity and cardiovascular disease [39], and sexual behavior [42]. Lotteries also take advantage of the motivational power and joy of games of chance [43], and are popular in Uganda where some lotteries are even implemented by cell phone.

The Behavioral Economic Incentives to Support HIV Treatment Adherence study (BEST) is examining these various issues and will address the significant problem of low ART adherence and lack of viral suppression through giving lottery incentives to HIV clients. In BEST, viral loads are the biological endpoints of the intervention. The study is investigating two different models for implementing lottery incentives, and one arm is based only on measures readily available in the clinic. This arm does not rely on adherence measurement devices, has a low cost, and hence, is readily implementable in a real-life clinic context. The suitability of these two intervention arms for take-up will be further assessed by a cost-effectiveness analysis. BEST will also measure the persistence of effects for up to 12 months post-intervention to contribute much-needed empirical data to the lively debate on the longer-term effects of financial rewards once they are withdrawn.

## Methods/design

### Study design

This study will use a three-armed randomized controlled trial (two intervention groups and one control group), with randomization at the individual level. The intervention will last for 24 months. See the SPIRIT checklist for a guide to the key items reported in this protocol (Additional file 1).

### Study sites

The study will be conducted at Mildmay Uganda, an NGO with headquarters in Uganda’s capital Kampala. Mildmay Uganda specializes in the provision of comprehensive HIV and AIDS prevention, care, and treatment services. Mildmay Uganda provides quality outpatient and inpatient HIV care and trains healthcare workers throughout Uganda and the region in the provision of such care. It also offers integrated health services and technical assistance to organizations and governments within and outside Uganda, as well as training and education courses for over 1500 professionals per year and has numerous ongoing research projects involving international researchers. Mildmay serves over 105,000 patients (15, 000 at the main site in Lweza and over 95,000 at supported health facilities in eight districts in the Central Region of Uganda). The main site has a well-trained and experienced team of clinicians and health workers, and a modern laboratory infrastructure with an ability to do virology and other tests. The Mildmay Uganda laboratory is accredited by the South African National Accreditation System under ISO 15,189:2012 for medical laboratories and also acts as a national backup laboratory for the Uganda Central Public Health Laboratories. Some of the services provided include HIV counseling and testing; pediatric and adult HIV prevention, treatment, and care services; sexual and reproductive health services; diagnostic (laboratory) services and radiology; rehabilitative services (nutrition, physiotherapy, and occupational therapy); safe male circumcision; ophthalmic services; and dental care. Of the 15,000 patients served at the main site in Lweza, 11% are children younger than 18, 65% are female, and 100% of all clients in care are on ART. Mildmay is one of a growing number of facilities with a well-established electronic medical records system in Uganda.

### Sampling and participants

We will recruit into the study 330 treatment-mature Mildmay clients. We have recruited patients at Mildmay in our previous studies without significant problems, and we have an ongoing collaboration with the medical records team that allows us to react swiftly to any problems. Indeed, we were able to recruit the target number of clients in less time than originally envisioned in previous studies.

Clinical records and the electronic database will be used to identify eligible clients. The inclusion criteria are described below. Clinic staff (rather than the study coordinator) will mine the clinic database for eligible clients, given that the client at that point has not yet consented to participating in the study. We will then recruit participants from the universe of eligible patients. This approach avoids many of the disadvantages of convenience sampling, such as the bias that may arise from sampling those most easily accessible.

Once eligible clients are identified in the database, the study team will use the next appointment as an opportunity for recruitment. Each day, the study coordinators will identify patients who are due for a visit that day and approach all those deemed eligible. Once a participant is located, the coordinator will initiate the pre-baseline visit by approaching them and inquiring whether they are interested in participating in an ongoing study. Respondents will also be informed that they will not lose their spot in the queue for seeing a provider. Patients who initially agree will be taken to a separate study room to verify their eligibility. We will then get consent from the patient to participate in the study. Once the participant gives consent, we will give them a medication event management system (MEMS) cap and instruct them to store all their HIV medication in a pill bottle with the MEMS cap attached. This MEMS cap will be used to record adherence, and participants will be asked to return with the MEMS cap at their next scheduled study visit in approximately 2–3 months, so the study team can retrieve their adherence data. These first 2–3 months of adherence data will be used as a baseline and the intervention will not begin before this follow-up visit.

### Inclusion criteria

Our study sample will be 330 HIV patients aged 18 and older who have been on ART at Mildmay for 2 years or longer to filter out clients who are at Mildmay only transiently and to target treatment-mature clients for whom it is likely that the motivational problems that are the target of our intervention are a significant barrier to adherence. They must have had recent adherence problems within the past 6 months based on clinical records (defined as showing lack of viral suppression, being sent to adherence counseling, or at disease stage 3 or 4 as per WHO guidelines). Our decision to enroll patients with adherence problems is based on our conceptual framework, which suggests that motivation is a key driver of adherence in treatment-mature HIV clients, as well as on meta-analysis data showing that HIV adherence interventions have a larger effect size for such clients (mean effect size 0.62) compared to effects on interventions applied to all patients (mean effect size 0.19), in general [44].

### Exclusion criteria

Clients who are not able to understand the consent and study procedures will be excluded, as will clients who speak neither English nor Luganda (the local language spoken by the large majority of people in and around Kampala). In our previous studies at Mildmay, only a small number of clients had to be excluded from study participation because of these criteria. Other exclusion criteria are whether they are in any other adherence-related study and whether they are not capable of using the MEMS cap regularly. During the first follow-up visit after recruitment (roughly 2 months after the participant is recruited), we will check the MEMS-cap usage data and exclude patients who have opened it on fewer than 30% of days, as one of the study requirements stated in the consent form is that they use the MEMS cap regularly. The participant will receive a transport refund for that visit but will be asked to return the MEMS cap and not come to the study offices anymore for study purposes. It is important to note that we interpret less than 30% adherence as inconsistent use of the MEMS cap, rather than simply poor adherence. Less than 30% true adherence is very rare. Therefore, we are not necessarily excluding those with very poor adherence, but rather those who realize after initially agreeing to use the MEMS cap that they are either not able or not willing to use it after having tested it. Other exclusion criteria include patients on any third-line treatment and those who come to the clinic outside regular working hours.

### Randomization

Randomization in a 1:1:1 ratio will occur after participants are recruited but before they complete a baseline survey to ensure that group assignment does not influence the answers given. We will stratify random assignment by age (under 25 or over 25), sex, marital status (married/cohabiting or unmarried), low CD4 count (below 200 or above 200), and viral load (detectable or undetectable). Stratified randomization is achieved by generating a separate block for each combination of covariates after identifying which clients fall into each block. We will then randomize treatment assignment within each block. We will use the *randtreat* package in Stata15 for the randomization procedure.

All clients recruited will complete the baseline survey approximately 2–3 months after recruitment. The client will be informed of their assignment to either one of the two intervention arms or the control group after completing the baseline survey.

Participants cannot be blinded to their treatment status and neither can interviewers. Interviewers are not blinded to treatment status when they read a MEMS cap. The data analyst who will conduct the impact analysis will be blinded to treatment assignment.

### Design

**Fig. 1:**
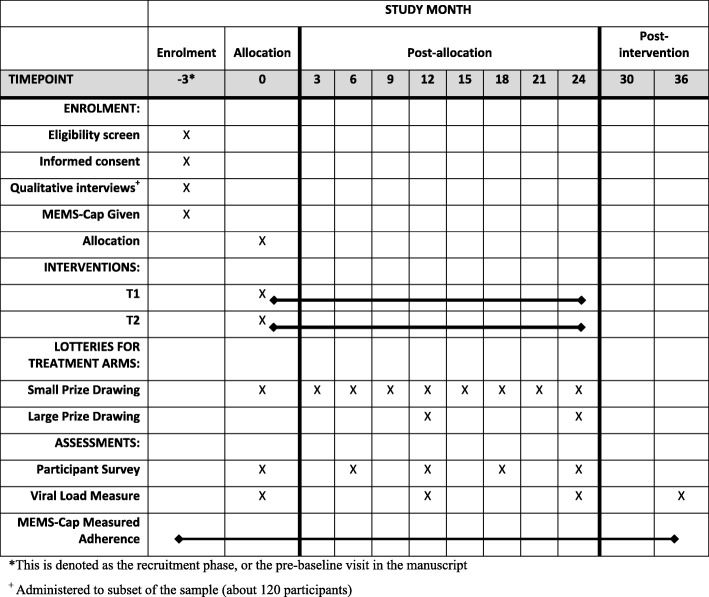
SPIRIT schedule of enrolment, allocation, interventions, and assessments. MEMS, medication event management system, T1 treatment group 1, T2 treatment group 2

The study has two intervention arms and a control arm. Both intervention arms will offer lottery-based incentives but with different conditions. We will collect treatment adherence data continuously for 2–3 months prior to the intervention, for 24 months after the intervention begins, and for 12 months after the intervention ends for all participants using MEMS caps. We will acquire routine viral load measures for all participants throughout the study, which will be recorded roughly every 12 months as per clinic and Ugandan Ministry of Health guidelines. We will also conduct a baseline survey and follow-up surveys every 6 months for 24 months for all participants. Figure 1 gives the timing of study activities.

### Procedures

#### Study interventions

There will be two intervention arms, both of which will use lottery-based incentives to encourage high adherence and viral suppression. In treatment group 1 (T1), clients will be eligible for quarterly lotteries with small prizes based on timely drug refills and annual lotteries with larger prizes if they demonstrate viral suppression. In treatment group 2 (T2), clients will be eligibility for quarterly lotteries with small prizes and annual lotteries with larger prizes based on high adherence as measured by the MEMS caps (all participants, including those in the control group, will receive MEMS caps). These interventions are described in more detail below.

#### Treatment group 1

For this group, lottery eligibility is based on timely drug refills at each clinic visit and achieving viral suppression.

##### Draws with small prizes at each scheduled clinic visit

Participants will be eligible to participate in prize draws whenever they come to the clinic as scheduled (roughly every 2–3 months). When a client assigned to T1 comes to the clinic, the study coordinator will check whether they have an appointment using the client’s clinic booklet and medical records. If the client does have an appointment, they are invited to draw a number out of a bag without looking. Each number corresponds to a different type of prize and they will have four prize options, as outlined in Table 1.

**Table 1:** Prizes awarded at each clinic visit for treatment group 1

Prize	Value (USD)	Win probability
No prize	0	0.25
Small water bottle	0.50	0.25
Large water bottle	1.00	0.25
Umbrella or coffee mug	1.50	0.25

Altogether, 75% of clients will win something in each draw, which is meant to minimize the discouragement of not winning anything as observed in our previous work [45]. Clients will win the smallest prize worth about .50 with probability of 0.25, the medium – sized prize with value of 1 with a probability of 0.25, and the largest prize valued at $1.50 with a probability of 0.25.

As in previous studies, we sometimes observed that there was a discrepancy between the scheduled appointment date in the client’s booklet and the medical records. We will let them enter into one draw a year when they arrive for what their booklet says is an appointment but which has been wrongly entered into the system, but it will be made clear to the client that this is a one-time exception only.

##### Annual prize draws

The T1 group is eligible for the annual lottery conditional on showing viral suppression in the annual assessment of viral load, which requires a long-term behavioral change. Approximately 1 year after recruitment, and after 2 years, each client will be due for their annual viral load test. Once the test results are available, the study coordinator will assess the client’s eligibility for the annual lottery based on whether they have a detectable viral load. For eligible clients, the draw for the larger prize (worth roughly USD 10 with a 10% chance of winning) will take place at the subsequent clinic visit. We will classify a viral load as undetectable using the cut-off HIV RNA < 200 copies/mL. The AIDS Clinical Trials Group defines virologic failure as a confirmed viral load > 200 copies/mL—a threshold that eliminates most cases of apparent viremia caused by viral load blips or assay variability.

#### Treatment group 2

For this group, lottery eligibility is based on measured adherence using the MEMS caps.

##### Prize draws at each scheduled clinic visit

When participants in this group learn about their treatment assignment, they will be informed that if at their next visit they have taken their medication on 90% or more of occasions when they should have taken it, they will be eligible for the lottery. They will also be informed that for the next 24 months, they will continue being eligible for the lottery if they continue to take their medication at or above this level.

Each time the client attends a scheduled clinic visit, the study coordinator will check the client’s MEMS cap. The study coordinator will fill out a short form to verify eligibility that among other things asks the client whether they sometimes pocket doses (i.e., whether they take out more than one dose for a given opening). In a previous study, this was a relatively common practice used by about 15% of the sample. For example, many clients working as boda-boda or moped taxi drivers indicated that they commonly took the morning pill at home from the pill bottle, but pocketed the evening dose and took it with them as they did not know whether they would be home in time to take the evening dose. To avoid unfairly punishing those clients who pocket doses (which would show up as missing doses in the MEMS software, reducing their measured adherence), we will base their eligibility on their adherence over the last 2 weeks and ask them specifically about which days they pocketed doses. This method was easily implemented in our previous studies and was performed within a reasonable time (under 5 minutes per client who indicated pocketing).

If the client is eligible (> 90% adherence), they are invited to participate in the prize draw as described for the T1 group above. Previously, we sometimes observed that there was a discrepancy between the MEMS data and the client’s evaluation of their adherence. Therefore, we will let them enter into one draw a year when they did not reach the adherence threshold (as a wild card) to avoid disappointing the participant and to avoid unfairly punishing clients for whom there may have been an error in the MEMS-cap data. However, it will be made clear to the client that this is a one-time exception.

##### Annual prize draws

We will conduct an annual draw for which eligibility is based on 90% adherence over the course of the year. The aim is to encourage a sustained change in health behavior. The study coordinator will check the participant’s record and determine if the client is eligible for each annual draw. We will create a separate file for each client so that this check is easily performed.

#### Control group: usual care

The control group will receive care as usual, including any adherence support mechanisms within usual care practices. All clients receive intensive ART orientations and adherence education when they first start ART, but there is no systematic adherence support mechanism for long-term ART clients. However, adherence counseling is available to patients who are referred to a counselor by their providers. All control group participants are encouraged to come to the study offices at each clinic visit to ensure that they spend an equal amount of time with the study coordinator as those in the intervention groups. During that visit, we check the MEMS device and ask the client if they have any difficulties in using it.

### MEMS-cap procedures

Medications will be distributed in pill bottles with MEMS caps to patients in all three groups (including the control group) at recruitment (i.e., prior to random assignment) to avoid spurious intervention effects due to the MEMS cap. These caps house a microelectronic chip that records the date and time of each bottle opening, enabling a precise and objective assessment of the timing of each dose and the patient’s pattern of pill-taking. The study coordinator will assist the patient in dispensing a supply of the medication into a bottle we provide with an attached cap, or they can simply put the MEMS cap onto the actual medication bottle if preferred by the patient. We will monitor adherence to only one antiretroviral medication as studies show that rates of adherence do not differ significantly across medications taken by an individual patient [46]. Participants will be asked to use the cap continuously throughout the study and return with the bottle and cap for each clinic visit. We will ask patients to notify the study coordinator if their provider changes their regimen during the study so that the data can be downloaded and the medication being monitored can be switched with the new one. Participants will be informed that the cap records when the bottle is opened. They will also be informed that these data will not be shared with clinicians. We will carefully discuss the restrictions associated with the electronic cap with the patient. Solutions to potential concerns that are acceptable to both the patient and the project will be provided. Once a participant has started using the cap, we will review the instructions and restrictions with the participant at each clinic visit.

### Study timeline

#### Recruitment

During recruitment (also called the pre-baseline visit), the client will be given a MEMS cap, which will be used to track baseline adherence and study eligibility for 2–3 months (depending on the date of the client’s next scheduled clinic visit). During the recruitment phase, clients will be consented to participate by study coordinators, and for some (about 120 in total), we will administer short, 30-min qualitative interviews (Section 2.13.2). Participants will be compensated with 20,000 USh for their time. They will be paid for completing questionnaires even if this does not happen on a scheduled clinic visit, such as when they come in late or early for a clinic appointment.

As appointment dates are readily available from the electronic medical records system, we can print out weekly lists of eligible clients together with when they are expected at the clinic and when they are scheduled for their next viral load test. We will enroll clients on the day of or within 3 months of their annual viral load test to synchronize the viral load testing with the study timeline. Doing so will allow us to benefit from existing clinic procedures and reduce the inconvenience and transport costs of the intervention for clients. Thus, participants will not take any additional viral load tests or attend additional clinic visits for the study. We will provide this list of participants to the study coordinator, who can then watch out for these clients in collaboration with the clinic receptionist.

We will consent and enroll 4–6 clients per day (some clients may refuse participation, though this has been rare in our previous studies) during the 6- to 9-month recruitment period. Based on the large clinic population and our previous experience, we expect to recruit 330 clients easily within a 6-month period but we have allowed sufficient extra time to accommodate for the clinic being closed on public holidays and for any other delays.

#### Baseline survey and randomization

When a client returns for their next scheduled clinic visit after recruitment (after about 2–3 months), we will conduct the baseline survey, reveal their treatment assignment to either the control or one of the two intervention groups, and begin the intervention. We will exclude all clients who did not open the MEMS cap for at least 30% of the days during the pre-baseline period as consistent use of the MEMS cap is essential for participation in the study. This second visit will (just as the first one) coincide with a scheduled clinic visit so that participants do not have to come to the clinic solely to participate in study activities.

The baseline survey contains information about:

1. demographics and socioeconomic status, including age, gender, education, relationship status, employment type and status, income, housing, economic shocks, and household composition
2. medical history, including history of opportunistic infections, health-seeking behavior, current medications and length of time on current drug regimen, and WHO HIV disease stage, some of which will be taken from their medical records
3. physical symptoms and side effects, for example, respondents will be asked how “bothersome” and “disruptive” (in separate items) any symptoms perceived to have been caused by their medication in the past month have been
4. reasons for non-adherence or failure to seek care; we will use an 11-item module regarding reasons for non-adherence developed by the AIDS Clinical Trials Group that we slightly modified to fit the Ugandan context that asks participants to indicate whether listed items were reasons for not taking their medication in the previous month or seeking care, such as “when the drugs make you feel bad,” or “when your daily routine is interrupted,” or “lacked resources”

We use the Intrinsic Motivation Inventory to examine participants’ subjective experiences of taking medications [47]. The survey will also collect information on behavioral economics biases, such as present bias or risk preferences.

#### Follow-up surveys

Follow-up surveys will be conducted at months 6, 12, 18, and 24. These assessments will allow us to collect several data points for each participant on mediators or moderators that we believe may be influenced by the intervention (e.g., cognitive and motivational factors). After the intervention is finished, we will continue to track the adherence of participants for 12 months to assess whether the intervention encourages adherence persistence after incentives are withdrawn.

#### Exit interviews

Altogether, 40 participants will be asked to attend a semi-structured exit interview after they have completed the 24-month intervention period. We will stratify the sample by gender and recruit people from each of the three study arms. The interview will address topics such as barriers and challenges related to ART adherence and attending scheduled clinic visits. Intervention participants will also be asked about barriers and challenges they may have faced during the intervention, and we will elicit feedback on their experience in the BEST program.

This semi-structured interview will be conducted by the study coordinator at Mildmay clinic on the day of a scheduled visit and will last approximately 30 min. Interviews will be audio recorded with the permission of the participant, and then translated and transcribed. The audio recording will be deleted once the transcription is complete. Hardcopy notes will be digitized and transferred to RAND via secure online transfer. Hardcopy notes will be stored at Mildmay and accessible only to study staff.

### Outcomes

#### Primary outcomes

We have one biological primary outcome and one behavioral primary outcome.

##### Viral suppression

Our biological primary outcome will be a binary indicator for whether the participant has an undetectable viral load. The AIDS Clinical Trials Group defines virologic failure as a confirmed viral load > 200 copies/mL. We will consider that a viral load is undetectable if it is below 200 copies/mL. Viral load is the primary measure used to assess the level of viral activity in a person’s blood, as well as their response to ART. Although other factors besides adherence contribute to viral load, having an undetectable viral load is widely considered a strong indicator of good ART adherence. Furthermore, given the limitations of measures of behavioral adherence, viral load is considered by some to be the best indicator of adherence, and at the very least a valuable complement to behavioral adherence measures. Viral load measurements are now– part of routine clinical care at Mildmay and results for participants will be taken from clinical records. Viral load is measured when a person receives a positive HIV test result, after 6 and 12 months, and then every 12 months. We will synchronize recruitment so that the viral load of all participants is assessed at baseline, around month 12, and around month 24.

##### Percentage of prescribed medication taken

Our behavioral primary outcome will be electronically monitored adherence. MEMS data will be collected continuously during the 24 months of the intervention period as well as for 12 months after the intervention ends, allowing us to investigate daily adherence and its timing. We will create a variable that captures the proportion of prescribed pills that were actually taken (i.e. number of actual bottle openings divided by the prescribed bottle openings).

### Sample size and power

We have calculated the size of effects that our sample will be able to detect with 80% power (two-tailed test) with regard to outcomes at months 12 and 24, and 10% attrition every year (we observed 5% attrition over 20 months in our previous studies so this is a conservative estimate). For the primary outcome of viral suppression, we use a conservative estimate of 70% of clients in the control group showing suppression, based on discussions with the Mildmay team. Our sample size of 110 participants in each of the three arms (total *n* = 330) will be able to detect a 7 percentage point difference for joint comparison of T1 and T2 against the control group at month 12, and about an 8.5 percentage point difference between the two intervention arms (a subgroup analysis). The corresponding differences at month 24 are 8 and 9 percentage points, respectively. These are considered small effect sizes (Cohen’s *d* of between .15 and .185), which we will be able to detect. For adherence, in our previous study we observed mean adherence rates of ~75% as measured by MEMS caps. Our sample size of 110 in each of the intervention arms and the control group will provide sufficient power to detect about a 6.5 percentage point difference in mean adherence between the two intervention arms (combined) and the control group. To test for differences in adherence between the two intervention arms, our study is powered to detect about a 7.5 percentage point effect as measured by MEMS caps. Again, this means we will be able to detect small effects. The corresponding differences we will be able to detect at month 24 are about 7 and 8 percentage points, respectively. Our main econometric models will include covariates that will improve precision. Therefore, these minimum detectable effects reported above are likely upper bounds.

### Data analysis

#### Quantitative data

Our primary analyses will be by intention to treat, with secondary analyses involving study completers only.

##### Estimating impact on viral suppression

We will use a logistic regression to compare the likelihood of viral suppression between the three study arms after year 1 and after year 2. We will estimate an unadjusted model and a model that includes prespecified covariates to adjust for baseline characteristics and improve precision. We will adjust for the following covariates: age, education, sex, baseline adherence, baseline WHO disease stage, baseline viral suppression, duration on ART, self-reported physical health, self-reported mental health, and HIV disclosure status.

Our main unadjusted model has the following form:

1 $$ \Pr \left({y}_i\right)=\mathrm{expit}\left({\beta}_0+{\beta}_1T{1}_i+{\beta}_2T{2}_i+{\epsilon}_i\right) $$

and our main adjusted model has the following form:

2 $$ \Pr \left({y}_i\right)=\mathrm{expit}\left({\beta}_0+{\beta}_1T{1}_i+{\beta}_2T{2}_i+{\boldsymbol{X}}_{\boldsymbol{i}}{\boldsymbol{\beta}}_{\mathbf{3}}+{\epsilon}_i\right) $$

where *y*_*i*_ is the probability of viral suppression of individual *i*, T1 is an indicator for treatment group 1, T2 is an indicator for treatment group 2, and *ϵ*_*i*_ is the idiosyncratic error. The coefficients of interest are *β*_1_ and *β*_2_. Equation 2 has the term ***X***_***i***_***β***_**3**_, which includes a vector of the prespecified covariates described above (***X***_***i***_) and the respective coefficients (***β***_**3**_). We will estimate the marginal difference (or risk difference) between each of the three study arms as the following:

- $\mathbbm{E}\left[{y}_i\right|T1=1\left]-\mathbbm{E}\left[{y}_i\right|\mathrm{Control}=1\right]$: Impact of treatment 1 on the outcome relative to the control group
- $\mathbbm{E}\left[{y}_i\right|T2=1\left]-\mathbbm{E}\left[{y}_i\right|\mathrm{Control}=1\right]$: Impact of treatment 2 on the outcome relative to the control group
- $\mathbbm{E}\left[{y}_i\right|T2=1\left]-\mathbbm{E}\left[{y}_i\right|T1=1\right]$: Impact of rewarding adherence relative to rewarding viral suppression

We will also estimate a model where we pool T1 and T2 (relative to the control group) to estimate the impact of lottery incentives more generally.

##### Estimating impact on adherence

To estimate the impact of our interventions on treatment adherence, we will use an ordinary least squares regression of the following forms:

Unadjusted:

3 $$ {y}_{it}={\beta}_0+{\beta}_1T{1}_{it}+{\beta}_2T{2}_{it}+{\lambda}_t+{\epsilon}_i $$

Adjusted:

4 $$ {y}_{it}={\beta}_0+{\beta}_1T{1}_{it}+{\beta}_2T{2}_{it}+{\lambda}_t+{\boldsymbol{X}}_{\boldsymbol{i}}\boldsymbol{\alpha} +{\epsilon}_i $$

where *y*_*it*_ represents the proportion of the prescribed bottle openings that were opened by individual *i* at time *t*. In this model, *β*_1_ represents the impact of T1 on the proportion of pills taken in a given month and *β*_2_ represents the impact of T2 on the proportion of pills taken in a given month. *λ*_*t*_ represents an indicator for each time period. Equation 4 includes the term ***X***_***i***_, which represents the same covariates as in Eq. 2.

##### Estimating impact on measured adherence over time

We will have a consecutive real-time measure of adherence for the duration of the study, which allows us to study adherence over time. To estimate the impact of the interventions on adherence over time, we will use the following ordinary least squares model:

5 $$ {y}_{it}={\beta}_0+\sum \limits_{t=1}^{24}{\lambda}_t\left(T{1}_{it}\times {\mathrm{Month}}_t\right)+\sum \limits_{t=2}^{24}{\delta}_t\left(T{2}_{it}\times {\mathrm{Month}}_t\right)+\sum \limits_{t=1}^{24}{\mathrm{Month}}_t+{\epsilon}_i $$

where *y*_*it*_ represents the proportion of the prescribed bottle openings that were actually opened by individual *i* in month *t*. In this model, the *λ*_*t*_’s represent the impact of T1 at study month *t* and the *δ*_*t*_’s represent the impact of T2 at study month *t*.

##### Subgroup analyses

We will conduct several subgroup analyses to provide insights into which types of clients are most likely to benefit from the intervention. We will assess intervention impact for the following subgroups:

1. Those with a strong vs. weak present bias
2. Those with a high vs. low baseline treatment adherence
3. Those with a high vs. low baseline intrinsic motivation to take treatment

##### Standard errors

We will estimate Huber–White robust standard errors in all analyses.

##### Adjusting for multiple hypotheses

We will adjust *p* values for multiple hypothesis testing for all secondary outcomes specified above (and any ad hoc outcomes we analyze ex post) using the free step-down re-sampling method to control the false discovery rate [48].

#### Qualitative data

We will conduct interviews with participants that examine: (1) their existing daily habits, (2) how they make decisions about their finances and health, (3) their decisions regarding their ART medications, and (4) how the medication influences the way they spend their money. The audio recordings of these interviews will be transcribed verbatim and translated from Luganda into English and stored on a secure data transfer website. The data will be entered into the software Dedoose and we will develop a structured codebook to identify a priori identified and emerging themes [49]. As is standard with a directed content analysis, the initial set of themes will be informed by existing issues identified in the peer-reviewed literature, complemented by our collective experiences with ART adherence in resource-poor settings.

#### Data management

Existing clinic identifiers will be used as unique study identification numbers during data collection. Consent forms will bear the name and signatures of study participants, but all other information (such as viral load tests, MEMS cap readings etc.) will be recorded using these unique clinic identifiers. Tablets used for data collection by study coordinators will be protected by passwords. The study team in Uganda (one team leader, two lead interviewers, and three supporting team members) will be in charge of collecting all data and carrying out the intervention. The Uganda team will transfer data weekly through a secure web portal (Kiteworks). The study team based in the U.S. will design data collection instruments and protocols, monitor qualitative and quantitative data for quality, and conduct all data analyses. Paper copies of consent forms will be stored and locked at the Mildmay RAND office in Kampala, and access will be granted to only key personnel and the principal investigator (PI). Any published material will not contain information that can be used to identify participants. There is no formal data monitoring committee since the trial was deemed minimal risk, but data monitoring will occur through weekly checks by the study team in the U.S.

#### Handling missing data and attrition

Missing data has been a minor issue in our previous studies with the same study population and outcomes. Attrition has been well under 10% per year. However, when subjects drop out, we will fit multiple logistic regression models to assess whether this dropout is random. If it is not, we will construct nonresponse weights using a logistic regression that correct for dropouts by assigning weights to continuing subjects that are inversely proportional to the predicted probability of the subjects’ continuing to the time period in question. Analyses will incorporate design effects from this weighting in the calculation of standard errors and tests of significance. In addition, we will perform sensitivity tests regarding changes in outcomes when excluding those with missing observations to give a fully transparent picture of the data.

#### Cost-effectiveness analysis

We will assess the relative cost-effectiveness of the two different ways of implementing the lottery incentives using standard methodologies [50]. We will estimate an incremental cost-effectiveness ratio (ICER), which is the incremental cost of the interventions divided by the incremental effectiveness of the interventions, for both primary outcomes:

6 $$ \mathrm{ICER}=\left({c}_2-{c}_1\right)/\left({e}_2-{e}_1\right) $$

where *c*_*i*_ is the per capita cost of the respective treatment group (1 or 2) and *e*_*i*_ is the number of participants with viral suppression or high adherence in the treatment group. The numerator is the incremental cost of T2 relative to T1, that is the cost of incentivizing the measured adherence relative to incentivizing the viral load (mostly the cost of the MEMS caps). The denominator is the number of additional people with an undetectable viral load or high adherence in T2 relative to T1. We will estimate confidence intervals for our ICERs using the upper and lower ends of the confidence intervals of our estimated effect sizes.

We will use a micro-costing approach to the analysis, i.e., we will carefully track all costs associated with implementing each intervention. Fixed costs, such as clinic rental costs and overheads, will be allocated as the fraction of time the premises are occupied by study personnel during the intervention (i.e., the number of hours per week that a room is used for intervention purposes). Costs in the analysis will be assessed using a clinic perspective, taking into account all intervention costs but not costs that accrue to the participant. Since the interventions take place at the same time as scheduled clinic visits, we expect few (if any) additional costs to the clients. Also, it would be difficult to value costs incurred accurately given the wide variation in patients’ opportunity costs, for example.

We will differentiate between development costs and ongoing costs. Development costs include personnel costs for training needed to implement the interventions but will exclude costs that are associated purely with the research activities (e.g., surveys). The costs of MEMS caps will be included as intervention costs for T2 (which requires them for measuring adherence) but not for T1 (which uses the caps only for measuring outcomes for study purposes but does not rely on them for implementing the prize draws). The evolution of the running costs will be tracked carefully to ascertain whether there are cost efficiencies over time. Within those, we will differentiate between the fixed costs of the intervention and the marginal cost of adding an additional client, which will provide information on the generalizability of the interventions to other settings.

## Discussion

### Potential impact and significance of the study

Treatment adherence is critical to the success of ART and is largely determined by behavior. This study will test novel behavioral interventions to improve ART adherence and has the potential to enhance HIV treatment and prevention significantly. An increasing number of people have been on ART for many years (i.e., they are treatment-mature), and they may lack motivation to adhere to their medication consistently. This study will be one of the first that we are aware of that tests an intervention designed to increase the motivation of treatment-mature clients for adherence. The findings of this study will provide unique insight into the underlying behavioral mechanisms that affect HIV treatment adherence, which could be exploited to improve adherence in a variety of settings.

In addition, this study will test an intervention that is readily and cheaply scalable. While previous work by the study team has demonstrated that lotteries based on MEMS caps can be effective at encouraging adherence, this study will compare the efficacy of using measured adherence vs. viral load to determine lottery eligibility. While MEMS caps are relatively costly, viral load testing is now part of routine clinical care in Uganda and therefore, is available at no additional cost. We will test whether directly incentivizing viral suppression is as effective as incentivizing electronically measured adherence, and also conduct a cost-effectiveness analysis. This will help guide whether incentivizing viral suppression should be scaled up or whether the extra cost of the MEMS caps is worth the potential extra benefits of directly incentivizing adherence.

### Reporting adverse events

While we are doing everything to avoid increasing the risk to study participants due to any study-related activity, we will be very careful in tracking any potential negative events experienced by any study participant. Adverse events relating to ancillary and post-trial care may encompass both physical and psychological harms. The study coordinator will be experienced and trained to recognize risks or crises that require referrals. Team members have established procedures and guidelines to respond to risk disclosures and crisis situations among participants. If there are indications during a study visit that a participant poses a risk of suicide or self-harm, the interviewer will stop the session and explain to the participant that they would like an on-site Mildmay mental health counselor to speak with the participant about the situation. The counselor will then assess the risk for potential harm and the appropriate action in terms of evaluating the client’s need for mental health services and notifying the appropriate authorities. This assessment should be done as soon as possible and before the client leaves the premises, to the extent possible. A serious adverse event report will be filed, if necessary. Everything will be done immediately if possible and certainly within 24 h.

Anything that looks like it could be an adverse event will be brought to the attention of the local and study PIs, since each case needs to be investigated. Any unexpected or serious adverse events that occur during the course of this investigation and follow-up period will be reported by telephone by the PI within the next business day to the study institutional review boards (IRBs) and the independent study monitor. The telephone report will be followed within 3 business days by a written report, which will contain: the subject’s ID, the title and date of the serious adverse event, and a narrative explanation (e.g., how the research staff was notified of the event, dates of consent, screening results for inclusion or exclusion, whether the participant participated in the intervention or was in the control group, dates and circumstances of the hospitalization or death, and the participant’s status at their last clinical or research contact). In consultation with the IRBs, the PI will address whether there is a need to redesign or amend the protocol, or to inform current and future subjects of a change in how risks are described (e.g., changes to the consent form or protocol).

Except for adverse events, we do not expect that the interventions will need to be discontinued for any reason. As per our IRB protocol, participants are free to withdraw from the study at their own discretion. Due to the low-risk nature of the intervention, there will be no interim analyses or stopping rules.

### Dissemination of results

Study findings will be disseminated to researchers and clinicians via peer-reviewed publications and conference presentations. Published papers will follow established guidelines for defining the level of contributions that warrant authorship. Our findings will be relevant to local Ugandan and global communities with an interest in understanding the underlying behavioral mechanisms that affect HIV treatment adherence. We will share these findings with senior officials at the Ministry of Health and at Mildmay Uganda, so they can formulate appropriate policies in line with the national recommendations on annual viral load screenings for HIV-positive patients.

### Limitations

This study has several limitations. First, the study will include only 330 patients from one clinic in Uganda. Although our sample size is well powered to detect clinically important effects, it is not clear that our results will extrapolate to other areas of Uganda or other countries. Second, while our study includes both large and small incentives, we are unable to disentangle the effect of these different incentive types and the extent to which there is a dose response with incentive size. The impact of offering different incentive types and amounts will require further research. We also cannot verify the longer-term effects of these incentives or whether any effects persist after the incentives are withdrawn beyond the 12-month period during which we will monitor the effects of the intervention. Third, although adherence is measured using MEMS caps, which is currently one of the most accurate ways to measure adherence, we cannot exclude the possibility that some participants will consciously manipulate the pill bottle openings to increase their chances of receiving the incentives. 

### Trial status

The trial registration number is NCT03494777. The study start date was 12 April 2018. The protocol reported here is dated 17 October 2018. Patient recruitment is currently underway. The primary completion date is 1 March 2022 and the study completion date is 1 July 2022.

## Supplementary information

**Additional file 1.** SPIRIT Checklist for BEST.

## Data Availability

External researchers interested in our data, survey instruments, and other research methodology and procedures will be able to obtain this information through collaborative agreements (e.g., data use agreements) with the PI and co-investigators, as required by the data-sharing policy of the National Institutes of Health.

## Abbreviations

ART: Antiretroviral treatment
BEST: Behavioral Economic Incentives to Support HIV Treatment Adherence study
HIV: Human immunodeficiency virus
ICER: Incremental cost-effectiveness ratio
IRB: Institutional review board
MEMS: Medication event management system
NGO: Non-governmental organization
PI: Principal investigator
T1: Treatment group 1
T2: Treatment group 2
